# Artificial Intelligence Applications for Thoracic Surgeons: “The Phenomenal Cosmic Powers of the Magic Lamp”

**DOI:** 10.3390/jcm13133750

**Published:** 2024-06-27

**Authors:** Giacomo Cusumano, Stefano D’Arrigo, Alberto Terminella, Filippo Lococo

**Affiliations:** 1 General Thoracic Surgery Unit, Azienda Ospedaliero Universitaria Policlinico “G. Rodolico-San Marco”, Via Santa Sofia 78, 95100 Catania, Italy; 2 Department of Surgery and Medical-Surgical Specialties, University of Catania, Via Santa Sofia 78, 95100 Catania, Italy; 3 Department of Computer, Control and Management Engineering, Università La Sapienza, 00185 Rome, Italy; 4 General Thoracic Surgery, Fondazione Policlinico Universitario A. Gemelli IRCCS, 00168 Rome, Italy; 5 Department of Thoracic Surgery, “Sacro Cuore”-Catholic University, 00168 Rome, Italy

**Keywords:** thoracic surgery, artificial intelligence, computer vision, lung cancer, robotics

## Abstract

In the digital age, artificial intelligence (AI) is emerging as a transformative force in various sectors, including medicine. This article explores the potential of AI, which is akin to the magical genie of Aladdin’s lamp, particularly within thoracic surgery and lung cancer management. It examines AI applications like machine learning and deep learning in achieving more precise diagnoses, preoperative risk assessment, and improved surgical outcomes. The challenges and advancements in AI integration, especially in computer vision and multi-modal models, are discussed alongside their impact on robotic surgery and operating room management. Despite its transformative potential, implementing AI in medicine faces challenges regarding data scarcity, interpretability issues, and ethical concerns. Collaboration between AI and medical communities is essential to address these challenges and unlock the full potential of AI in revolutionizing clinical practice. This article underscores the importance of further research and interdisciplinary collaboration to ensure the safe and effective deployment of AI in real-world clinical settings.

## 1. Introduction

In the digital age, artificial intelligence (AI) is emerging as a powerful tool poised to revolutionize various sectors, including medicine [1]. The transformative potential of artificial intelligence today is akin to the phenomenal cosmic powers of the genie contained within the enchanted lamp of Aladdin. Like a magic genie, the applications of machine learning and deep learning techniques promise to grant the wishes of clinicians and surgeons, yielding significant advancements in critical areas such as diagnosis, prognosis, treatment planning, and pharmaceutical research. In the context of lung cancer, the main subject of care for thoracic surgeons, the impact of AI has been particularly profound. The field has undergone a substantial transformation, characterized by increasingly intricate diagnostic processes and complex therapeutic protocols. These advancements have integrated various omics domains—such as genomics, proteomics, and metabolomics—facilitating a shift toward a more personalized and preventive healthcare approach. This integration allows for a more precise understanding of the molecular underpinnings of lung cancer, leading to more targeted and effective treatments. AI-driven tools are now capable of analyzing vast datasets that include genetic, clinical, and imaging information, providing a more comprehensive picture of the patient’s condition. This holistic approach not only enhances the accuracy of diagnoses but also allows for the development of personalized treatment plans that are tailored to the specific genetic and molecular profile of the individual’s cancer. Furthermore, AI is revolutionizing prognosis by enabling the prediction of disease progression and patient outcomes with greater accuracy. In addition to these advancements, AI is also transforming treatment planning and pharmaceutical research. Machine learning algorithms can identify potential drug candidates by analyzing the biological pathways involved in lung cancer, accelerating the drug discovery process. AI-powered systems can also optimize treatment protocols by predicting how different patients will respond to various therapies, thereby improving the effectiveness of treatments and reducing adverse effects.

Numerous applications in thoracic surgery can be also identified, but drawing inspiration from the Aladdin tale, the three most important wishes of the thoracic surgeon might be achieving an accurate preoperative diagnosis of lung lesions; evaluating and mitigating preoperative risks; and enhancing surgical performance by choosing a personalized surgical approach. While the potential of these methodologies may seem unlimited, a multitude of unanswered questions regarding their true effectiveness have emerged. Of particular concern are the control systems within the learning mechanisms of neural networks, as well as the short- and long-term supervision of their associated outcomes. Significant risks can indeed arise regarding the quality of data input, the depersonalization of care, and the tendency to focus treatment on the disease rather than the patient as a whole. Additionally, we must not overlook the potential dangers associated with access to and the manipulation of sensitive data, as well as the risk of discrimination.

Large Language Models (LLMs) and multi-modal models are emerging as promising instruments to enhance diagnostic precision, operational efficiency, and personalized care.

## 2. LLMs and Chatbots

LLMs, exemplified by models such as GPT-3 [2] and LLaMA [3], are advanced artificial intelligence systems proficient in processing and generating text in a highly sophisticated manner. These models have diverse applications in the medical field. For instance, they can analyze medical records and reports to identify patterns and anomalies indicative of pathology, thereby aiding in the early detection and diagnosis of diseases. Additionally, LLMs can produce concise and comprehensible medical reports for patients and other healthcare professionals, streamlining the communication process and ensuring clarity in conveying medical information. Furthermore, they can be used to create chatbots capable of automatically delivering information and support to patients, providing a reliable source of guidance and assistance without the need for constant human intervention. In the wake of the success of LLMs in various scientific domains, a significant area of research has been focused on applying these models in the medical and pharmaceutical fields [4]. For instance, M. Sallam [5] has examined the applicability of ChatGPT in the biomedical sector, assessing its potential in scientific outputs, clinical case analysis, and aiding in diagnostic processes. However, this research also highlights several limitations and associated risks. These include ethical and copyright concerns regarding the data used to train such models, as well as the potential for inaccuracies in responses, occasionally lacking real or scientific validation, a phenomenon often referred to as “hallucination” [6,7]. Concerning the matter of data, machine learning techniques have fundamentally transformed traditional programming methods, where algorithms were traditionally coded with predefined rules to achieve specific outcomes using data. Human programmers provided the knowledge necessary for these rule-based systems. In contrast, the new paradigm involves models learning rules directly from the data they are trained on. In this updated approach, the role of the programmer resembles that of a teacher guiding a student through the learning process—providing essential information and assessing their preparedness at the conclusion. This approach can be aptly described as “programming with data”. It is crucial to note that an AI model trained on biased, unrepresentative, or poor-quality data will yield similarly inadequate results. Therefore, meticulous selection and preparation of data for training and validating AI systems are of paramount importance. To enhance reliability in the medical domain, several recent studies have subjected cutting-edge LLMs to additional training phases using strictly medical data, including publications sourced from the PubMed database [8,9,10]. Researchers at Google Research and Google DeepMind have introduced Med-PaLM [11], a model designed to process textual data, which has demonstrated a promising performance across various benchmarks. Despite these advancements, the accuracy of Med-PaLM still falls short when compared to human experts’ judgment, indicating that while LLMs hold great potential, there is still considerable work needed to fully harness their capabilities in the medical field.

## 3. Computer Vision and Multi-Modal Models

Multi-modal models integrate a wide array of data types, including images, text, and audio, to comprehensively understand a patient’s medical condition. Their applications range from analyzing radiological and biomedical images to identifying pathologies with greater precision, developing patient monitoring systems for the early detection of signs of clinical deterioration, and creating virtual assistants capable of natural and intuitive interactions with patients. Given that biomedical data often exist in non-textual formats like images, scans, or temporal sequences, several studies have proposed computer vision models trained on medical data. Some focus areas include tumor detection and identifying other pathologies from X-ray scans, ultrasounds, or magnetic resonance imaging [12,13]. Traditional computer vision algorithms relied on extracting a set of low- or high-level features from images or videos (e.g., points of interest, color intensity, edges, etc.), which were then used to train supervised learning models like support vector machines (SVMs) [14], random forests [15], or others for tasks such as object recognition or image classification. Others have concentrated on developing models capable of processing data in various formats, including multi-modal ones [16]. Recent advancements, particularly through deep learning methods like convolutional neural networks (CNNs), have significantly advanced the field of computer vision [17]. These methodologies are pivotal in radiomics, where increasing evidence shows that they enable the quantitative characterization of tumors for disease characterization or outcome prediction [18], outperforming, by far, human operators and any other previous related technologies in image recognition and analysis. In this framework, radiomics is an emerging and rapidly developing field that integrates knowledge from radiology, oncology, and computer science, emphasizing the integration of medicine and engineering [19]. Increasing evidence shows that radiomics can be used for the quantitative characterization of tumors for tasks such as disease characterization or outcome prediction, which constitutes an important research direction in medical applications [20]. Prior to CNNs, improvements in image classification, segmentation, and object detection were marginal and incremental. The introduction of CNNs has revolutionized this field. Furthermore, the advent of the transformer architecture [21] and its application to vision tasks [22] has enabled deep learning models to take a step toward accurate performance [23,24]. The extension of these models to jointly process multiple input types has made them applicable to several real-world scenarios, and thereby increasingly appealing for the medical field. For instance, T. Tu and colleagues at Google DeepMind recently introduced a multi-modal version of Med-PaLM, known as Med-PaLM M [25], a multi-modal variant that simultaneously processes textual and image data, markedly enhancing diagnostic accuracy and clinical case recognition comparable to human experts.

The integration of imaging data with histological and genetic characteristics forms the foundation of what I define as “personalized medicine”. However, there exists a not-insignificant risk that the focus is not on the individual (the tumor’s owner), but rather on the lesion itself (including histology, genomics, etc.). The distinction between targeting the lesion versus the individual is pivotal. While AI can undoubtedly enhance our comprehension of lesions through histology and genomics, it is vital to bear in mind that the ultimate objective is to enhance patient outcomes. The concern raised regarding the risk that the primary focus might be on the lesion rather than the individual underscores a critical differentiation. While AI can advance our understanding of pathological conditions through histological and genomic analysis, it is equally essential to uphold the ultimate goal of improving outcomes for patients holistically.

## 4. The First Wish

Delving specifically into the realm of thoracic surgery, the foremost wish revolves around achieving a precise and reliable preoperative diagnosis in the shortest possible timeframe. Early detection of lung malignancies is paramount for improving survival rates. Consequently, the approach to managing lung nodules has been significantly shaped by the implementation of AI, particularly through computer vision and multi-modal models. The high variability among radiologists in detecting lung nodules and the elevated false-positive rate in screening programs as reported in the National Lung Screening Trial [26] underscores the critical need for tools that can assist radiologists in identifying nodules, measuring them accurately, stratifying risk, and monitoring their progression. Computer-aided diagnosis (CAD) techniques were developed in the 1970s to improve the efficacy of chest radiography for nodule detection [27]. These computer algorithms in CAD systems can enhance the diagnosis of a range of medical conditions, utilizing computer-aided detection systems to identify abnormalities or lesions in medical images and computer-aided diagnosis systems to aid in their interpretation. Such systems have the potential to enhance the precision and speed of medical diagnosis, particularly in scenarios where human interpretation may be constrained or susceptible to errors. Among other supervised machine learning algorithms, SVMs and random forests are extensively employed in the diagnosis of lung diseases, with SVMs demonstrating efficacy in improving diagnostic efficiency [28] and random forests proving effective in the classification of non-small-cell lung cancer [29].

Despite the advances brought about by CAD systems in detection and efficiency, their widespread adoption in clinical settings is hindered by their high false-positive rate [30]. The advent of deep learning techniques, however, has garnered considerable attention owing to their capacity to enhance diagnostic accuracy. CNNs, introduced by Krizhevsky et al., demonstrated their superiority in detecting lung nodules compared to traditional CAD methods [31]. CNNs excel in learning features directly from images, thereby reducing false-positive rates and potentially averting unnecessary follow-up procedures [32,33]. Unlike CAD systems, the innovation of CNNs lies in their ability to learn from verified data and autonomously determine previously unknown features, thereby maximizing classification with limited direct supervision [34]. Consequently, this architecture of feature extraction through convolutional layers has proven highly effective for tasks such as image classification and segmentation.

## 5. The Second Wish

The second of the three wishes of thoracic surgeons centers on using AI to forecast and mitigate perioperative risk [35]. Preoperative assessment is a vital component of thoracic surgery, as it helps to evaluate and optimize a patient’s condition before undergoing surgery. The evaluation of respiratory function and the calculation of the predicted postoperative residual lung function, together with managing existing conditions such as COPD, heart disease, or diabetes preoperatively, is the cornerstone of ensuring the best possible outcomes. This comprehensive assessment and the consequent stratification of perioperative risk provide an opportunity to educate patients about the procedure, potential risks, and expected outcomes, ensuring informed consent. In recent decades, artificial intelligence has gathered substantial attention in the realm of preoperative risk assessment, resulting in the proliferation of various machine learning algorithms aimed at predicting the likelihood of major complications and mortality following surgery [36]. Within this sphere, AI-driven technologies have shown promising outcomes, providing valuable assistance in the decision-making process and in formulating comprehensive risk assessments, even in cases of major lung resection. Given the heightened morbidity associated with such procedures, the thorough evaluation of patients to determine individual risks and prognoses is of the utmost importance [37]. A variety of algorithms have been proposed for this purpose. Among these, some, employing diverse models of probabilistic neural networks, have successfully estimated postoperative prognosis following lung resection [38] and cardio-respiratory morbidity subsequent to lung resection for non-small-cell lung cancer (NSCLC) [39]. Others have achieved encouraging results by devising a model capable of delineating the risk of cardiac and pulmonary complications during the postoperative phase in patients undergoing anatomical lung resection through an innovative machine learning approach known as XGBOOST [40]. Additionally, researchers have managed to predict the onset of respiratory failure after lobectomy by identifying risk factors and introducing two machine learning-based techniques for predicting respiratory failure, supporting both quality review processes and clinical decision-making [37]. Machine learning algorithms hold promise in optimizing risk assessment for individual patients, enhancing the efficacy of preoperative evaluations, recommending suitable therapeutic strategies, and facilitating communication with patients and their families.

## 6. The Third Wish

The third and final wish involves the integration of AI-based technologies in the operating room environment. Although their current role is somewhat restricted, there is optimism that these technologies will progressively contribute to enhancing surgical precision and safety, facilitating intraoperative decision-making, and predicting postoperative outcomes in the near future. Recent evidence indicates that sublobar lung resections can achieve outcomes comparable to lobectomies for early-stage tumors [41]. The introduction of screening methods has improved the detection of early-stage diseases, leading to an increased use of more limited resections. In this context, a thorough preoperative understanding of pulmonary anatomy is crucial for precise surgical planning and appropriate case selection. Identifying intersegmental divisions via computed tomography is extremely challenging. For the preoperative planning of segmentectomies, virtual reality and artificial intelligence can facilitate three-dimensional visualization of the complex anatomy of pulmonary segmental divisions, vascular arborization, and bronchial structures. Some studies, such as the pilot study by Sadeghi and colleagues, have demonstrated the effectiveness of this technology [42]. Surgeons can use it preoperatively to gain a better understanding of the patient’s anatomy, aiding in the planning of segmentectomy.

One of the most promising applications of AI lies in robotic surgery, particularly in the field of thoracic surgery and the treatment of lung cancer, where it has demonstrated reductions in hospital stay duration and postoperative complications. Despite being associated with AI, robotic-assisted surgery does not yet incorporate AI-based technology; it still requires constant supervision by human surgeons. Though initially met with skepticism regarding the feasibility of fully automated surgery, advancements in robotic surgery have sparked interest in exploring the potential for autonomous actions in procedures such as interventional radiology, endoscopy, and various types of surgery [43].

Robotic systems offer enhanced three-dimensional (3D) visualization and magnification, along with effector instruments capable of wide-ranging motion, thereby augmenting surgical dexterity during procedures. Nonetheless, the absence of tactile feedback poses a challenge to achieving optimal surgical outcomes. Research efforts are underway to explore possibilities such as robots learning to manage tension on sutures and anastomoses or providing feedback on tissue compression through auditory cues. Furthermore, in striving for greater autonomy in robots, perhaps the focus should shift from haptics as perceived by humans to haptics as perceived by robots and computers [44].

The complexities involved in translating machine learning into effective and safe actions in humans are evident. AI applications require the storage of extensive video recordings of surgical procedures, necessitating meticulous data collection, preparation, and annotation, which must become integral to future medical practice. This underscores the importance of interdisciplinary collaboration between AI and medical communities [45]. Moreover, while AI models have demonstrated comparable or superior performance to humans, the complexity of these models makes it difficult to interpret and understand how they arrive at their decisions, which has led to the concept of AI models as “black boxes” [46]. Another major concern is the generalizability of these models across diverse patient populations, which could potentially be addressed through the development of continuous cloud-based learning systems for the real-time delivery of clinical records and ongoing refinement of training models. This approach would ensure the machine-independent reproducibility of the models [47].

Finally, AI-based technologies are influencing two other areas within the operating room: education and the improvement of management processes. AI applications hold promise for advancing precision surgery and surgical training, with machine learning algorithms being proposed for the accurate assessment of surgical skills, providing feedback during learning curves and periodic evaluations [48,49]. Despite the clear advantages, robotic surgery is associated with prolonged procedural times and substantial costs, necessitating precise scheduling of surgical procedures. Improved and optimized surgical procedure planning, particularly in robotic surgery, can be achieved through AI algorithms capable of precise procedure planning, accurate prediction of case durations, and the identification of surgeries at a high risk of cancellation. Ultimately, the use of machine learning models could significantly enhance operating room efficiency, leading to cost savings and optimal resource utilization, which are particularly important given the challenges to healthcare system sustainability posed by the high costs of new technologies.

Healthcare settings are increasingly witnessing the integration of advanced technologies. In perioperative medicine, the implementation of machine learning algorithms holds the potential to drive a multidisciplinary approach, particularly in preoperative assessment, risk stratification, and postoperative outcomes. Table 1 summarizes the potential applications of AI in various fields of thoracic surgery. 

## 7. AI Challenges and Dangers

Despite its potential, integrating AI into medicine presents significant challenges, particularly concerning data scarcity, interpretability, and the risk of bias. Leveraging AI techniques offers researchers and physicians the ability to manage the complexity of quantitative big data-related features. Therefore, it is crucial for oncologists, radiologists, and surgeons to continue incorporating machine learning tools into the clinical care continuum of NSCLC and to actively participate in the digital revolution that has already transformed the business and technology sectors. However, it is imperative to emphasize that research in this field is still in its early stages, and numerous challenges must be overcome before AI can be safely and effectively implemented autonomously in real-world clinical settings. Alongside the technical constraints highlighted, ethical and legal concerns demand careful consideration [50]. In particular, while AI in medicine holds much promise, the rights of individuals must be meticulously protected, particularly regarding privacy. Great attention must be paid to the protection of privacy, which has become increasingly apparent in the healthcare sector, where advancements must navigate the safeguarding of personal and highly sensitive information. Consequently, some scientific societies have developed specific guidelines to address this issue [51], aiming to balance between progress and privacy [52]. Finally, the gap between human intuition, judgment, and experience (the human element) and the analytical capabilities of AI (AI-driven factor analysis) is indeed a critical concern. This gap can lead to misunderstandings, misinterpretations, and a lack of trust in AI systems by humans. Additionally, there is a high risk of inefficiencies and errors whenever AI outputs are not adequately contextualized by human oversight. Furthermore, the indiscriminate and uncritical application of AI in therapeutic decision-making can increase risks associated with the human element, potentially causing inappropriate and even discriminatory medical decisions. This risk is particularly pronounced when AI models are trained on biased or unrepresentative data. Addressing this disparity requires enhancing AI transparency, interpretability, and fostering collaboration between AI systems and human experts. This approach might be useful to ensure that AI tools augment rather than replace human decision-making processes.

## 8. Conclusions

The magical genie of AI promises to revolutionize medicine as we know it today. Although its potential is vast, many of its powers are still hidden, and they may be able to fulfill dreams that were previously unattainable. Nonetheless, there are numerous challenges, risks, and pitfalls on this journey that must be meticulously navigated before AI can be safely and effectively utilized in autonomous clinical settings. Addressing these issues requires a concerted effort from the entire medical community, combined with robust ethical and legal frameworks to ensure that the integration of AI enhances patient care while protecting individual rights.

## Figures and Tables

**Table 1:** The various applications of AI in thoracic surgery highlight specific AI techniques and their impacts on clinical practice, from diagnosis and risk assessment to surgical procedures and training.

Area of Application	Description
Preoperative Diagnosis	Computer vision and multi-modal models enhance the early detection of lung malignancies by accurately identifying and measuring lung nodules. CAD systems and CNNs improve diagnostic accuracy and reduce false-positive rates, which are crucial for improving survival rates.
Perioperative Risk Assessment	AI-driven technologies help predict complications and mortality risks post-surgery. Algorithms like probabilistic neural networks and XGBOOST model cardio-respiratory morbidity and predict the onset of respiratory failure, supporting clinical decision-making and enhancing patient risk stratification.
Operating Room Environment	AI contributes to enhancing surgical precision, safety, and decision-making in robotic-assisted surgery. Machine learning algorithms facilitate the precise assessment of surgical skills and optimization of surgical planning.
Education and Management Processes	AI technologies provide educational support and feedback during surgical training. They also have the potential to improve operating room efficiency, scheduling, and overall resource utilization in healthcare settings and enhance the cost-effectiveness of patient care.

CAD, computer-aided diagnosis; CNNs, convolutional neural networks.
